# Concomitant underexpression of TGFBR2 and overexpression of hTERT are associated with poor prognosis in cervical cancer

**DOI:** 10.1038/srep41670

**Published:** 2017-02-14

**Authors:** Hui Yang, Hongyan Zhang, Yahua Zhong, Qiaoli Wang, Lei Yang, Hong Kang, Xiaojia Gao, Haijun Yu, Conghua Xie, Fuxiang Zhou, Yunfeng Zhou

**Affiliations:** 1 Hubei Key Laboratory of Tumor Biological Behavior, Hubei Cancer Clinical Study Center, Zhongnan Hospital, Wuhan University, Wuhan, China; 2 Department of Radiation Oncology & Medical Oncology, Zhongnan Hospital, Wuhan University, Wuhan, P.R. China

## Abstract

The human telomerase reverse transcriptase (hTERT) is highly expressed in a variety of tumors. The transforming growth factor beta receptor type II (TGFBR2) is a downstream protein of transforming growth factor beta (TGF-β) which suppresses telomerase activity. However, the relevance of survival to the expression of TGFBR2, hTERT or TGFBR2/hTERT has not been previously investigated in cervical cancer tissues. Our study showed that patients with low level of TGFBR2 were associated with poor prognosis (HR = 1.704, P = 0.021), but no significant relevance between hTERT expression and survival (HR = 1.390, P = 0.181). However, a combination of low level of TGFBR2 and high level of hTERT was associated with a worse survival (HR = 1.892, P = 0.020), which had higher impact of hazard ratio (HR) on the overall survival (OS) than the low TGFBR2 expression alone. Knockdown of *TGFBR2* expression by shRNA in Hela cells increased cell proliferation, cell invasion, G1/S transition and telomere homeostasis but decreased cell apoptosis. Overexpressing TGFBR2 and inhibiting hTERT suppressed Hela cell growth. These results would lead us to further explore whether a phenotype of TGFBR2^low^/hTERT^high^ could be considered as a predictor of poor prognosis, and whether simultaneous use of TGFBR2 agonist and hTERT inhibitor could be developed as a therapeutic strategy.

Cervical cancer is the second most common malignant tumor in women. In 2012, there are about 530,000 new cases of cervical cancer worldwide, of which 85% occur in developing countries^1^. About 275,000 women die of cervical cancer yearly, and 88% of deaths occurred in developing countries. In China, more than 75,000 new cases with 34,000 deaths annually^2^. Although the screening rate of human papilloma virus (HPV) is increased, the incidence of cervical cancer remains high. Thus, discovering reliable biomarkers is crucial for the development of potential therapeutic strategy for treating cervical cancer.

Telomere is a hallmark of cancer. Telomerase, including human telomerase reverse transcriptase (hTERT) and human telomerase RNA (hTR), is a ribonucleoprotein polymerase that retains telomere ends by addition of the telomere repeat sequence TTAGGG^3^. Activation of telomerase is detected in cancer cells but rarely in normal cells. Both hTR and hTERT are highly expressed and linked to high risk for a variety of cancers^4^, such as esophageal^4^,^5^, stomach carcinoma^6^,^7^ and human soft tissue sarcomas^8^. In cervical cancer, nevertheless, *hTERT* but not *hTR*, showed significant difference between normal cervices and cervical cancers^9^,^10^. Moreover, hTERT inhibitor (AZT or BIBR1532) or knockdown of *hTERT* by siRNA inhibit cell growth or enhances chemoradiotherapeutic sensitivity in Hela cells^11^,^12^,^13^,^14^. These findings suggest that hTERT might be a therapeutic target in cervical cancer. However, the role of hTERT in the prognosis of cervical cancer is still under debate as the hTERT expression is found to not associate with survival^15^.

Transforming growth factor beta receptor type II (TGFBR2), as the members of the TGF-β/Smad pathway, is a cancer suppressor. Underexpression or mutation of TGFBR2 is found in a number of cancers except cervical cancer^16^. TGFBR2 down-regulation promotes the development of invasive squamous cell carcinoma in intraepithelial neoplasia in the prostate and in the forestomach^17^. Moreover, previous *in vivo* study showed that mice lacking *TGFBR2* expression led to carcinoma in anal or genital^18^, indicating that the loss of TGFBR2 expression promotes carcinogenesis in epithelia^19^. *In vitro* and *in vivo* studies showed that soluble TGFBR2 inhibited cell growth, migration, invasion and metastasis in pancreatic and breast cancer^20^. Furthermore, farnesyltransferase inhibitor (L-744, 832) enhances radiation sensitivity via regulating TGFBR2 expression in pancreatic cancer cell line^21^. Thus, TGFBR2 is a cancer suppressor and a potential therapeutic target in cervical cancer. However, few studies have been focused on the role of TGFBR2 in the diagnosis and prognosis of cervical cancer.

TGF-β binds to TGFBR2 to transduce signal into cytoplasm^16^. Recent studies reveal that TGF-β represses *hTERT* gene expression and induces cell apoptosis and cell cycle arrest that is dependent on telomerase^22^. However, hTERT also regulates cell migration or tumorigenesis independent of telomerase^23^,^24^. Thus, using the altered expression of TGFBR2 and hTERT to predict the prognostic of cervical cancer may have clinical significance. Because this strategy might not only strengthen the telomerase dependent pathway of hTERT to control tumor, but also fill in the gaps which are produced by TGFBR2 that controls tumor only dependent on telomerase.

In this study, we investigated the correlation of hTERT expression with survival and examined the possible role of TGFBR2 in the diagnosis and prognosis of cervical cancer. We also tested whether the dual TGFBR2/hTERT tumor genotype is a more reliable predictor for the prognosis of cervical cancer than TGFBR2 or hTERT alone.

## Results

### Tissue microarray construction and immunohistochemical findings

**Figure 1:**
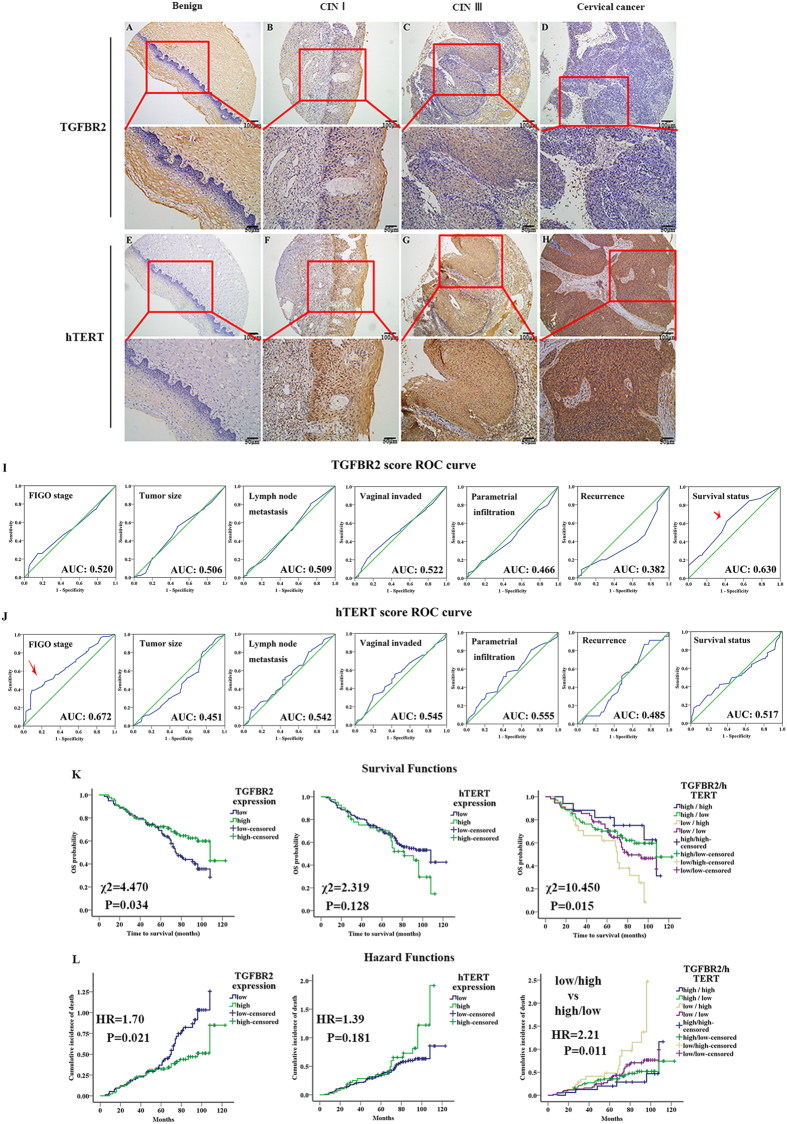
Immunohistochemical staining of TGFBR2 and hTERT protein in different cervical leisions. (**A**,**B**,**C**,**D**) TGFBR2 staining in benign lesion chronic cervicitis epithelium, CIN-I, CIN-III and cervical carcinoma samples, respectively. (**E**,**F**,**G**,**H**) hTERT staining in benign lesion chronic cervicitis epithelium, CIN-I, CIN-III and cervical carcinoma samples, respectively. (**I**) Receiver operating characteristic (ROC) curve analysis was used to define the high expression of TGFBR2. The specificity for each outcome of TGFBR2 staining was plotted: FIGO stage (P = 0.839), Tumor size (P = 0.714), Lymph node metastasis (P = 0.019), Vaginal invaded (P = 0.786), Parametrial infiltration (P = 0.069), Recurrence (P = 0.741), Survival status (P = 0.008). (**J**) (ROC) curve analysis was used to define the high expression of hTERT. The specificity for each outcome of hTERT staining was plotted: FIGO stage (P = 0.021), Tumor size (P = 0.001), Lymph node metastasis (P = 0.331), Vaginal invaded (P = 0.744), Parametrial infiltration (P = 0.967), Recurrence (P = 0.324), Survival status (P = 0.005). (**K**) Kaplan-Meier estimates for overall survival according to TGFBR2, hTERT and dual TGFBR2/hTERT expression, respectively. (**L**) Cox proportional hazards estimates for overall survival among patients with TGFBR2^high^ vs. TGFBR2^low^, hTERT^high^ vs. hTERT^low^, and dual TGFBR2/TERT group, respectively.

Tissue microarray and immunostaining were constructed successfully (see Supplementary Fig. S1). As shown in Fig. 1A–D, TGFBR2 was expressed in all three groups, primarily as cytoplasmic and membranous staining. The hTERT expression was detected in a nuclear and cytoplasmic staining pattern, whereas the subcellular localization of hTERT staining was in the nuclear pattern in normal cervical tissue (Fig. 1E), in both cytoplasmic and nuclear patterns in cervical intraepithelial neoplasia (CIN) tissues (Fig. 1F–G), and predominantly in the cytoplasmic in cancer tissues despite a nuclear component (Fig. 1H).

### Correlations between expression of TGFBR2, hTERT and progression of cervical lesions

As shown in Table 1, TGFBR2 manifested a moderate or strong staining in normal tissues, with low immunoreactivity in CIN-II and CIN-III tissues. The TGFBR2 expression was low or undetectable in most cervical cancer tissues. The proportion of undetectable staining in normal and CIN-I tissues was less than that in CIN-II, CIN-III, and cervical cancer tissues. The expression rate (1/11, 9.2%) of TGFBR2 was the highest in normal tissues. As summarized in Table 1, when the five specimen grades (i.e, chronic cervicitis, CIN-I, CIN-II, CIN-III, and cervical cancer) were compared with the frequency of moderate and strong staining, the TGFBR2 expression was found to gradually decrease in the following order: chronic cervicitis (5/11, 45.5%) >CIN-I (14/51, 27.4%) >CIN-II (4/19, 21.1%) >CIN-III (2/27, 7.4%) >cervical cancer (5/164, 3.0%) (p = 0.000, p < 0.05). In addition, the difference between the chronic cervicitis group and the CIN-III group (or cervical cancer group) was significant (p = 0.034, p = 0.001; p < 0.05). Similar difference was observed between CIN-I and cervical cancer (p = 0.000, p < 0.05).

The hTERT expression did not have strong staining in the chronic cervicitis group. Table 2 showed that when the five specimen grades (i.e., chronic cervicitis, CIN-I, CIN-II, CIN-III, and cervical cancer) were compared with the frequency of negative staining, the rate of hTERT positive expression generally increased gradually with histopathological grade in the following order: chronic cervicitis (9/11, 81.8%) < CIN- I- III (92/97, 94.8%) < cervical cancer (156/164, 95.1%) (p = 0.000, p < 0.05). In addition, significant difference was found between the chronic cervicitis group and CIN-III or the cervical cancer group (P = 0.001, P = 0.014; P < 0.05), with the same as the CIN-I versus cervical cancer (P = 0.004, P < 0.05).

### Selection of cut-off score for high expression of TGFBR2 or hTERT

To understand the relationship between TGFBR2 (or hTERT expression) and clinicopathologic features, a ROC curve analysis was used to define a cut-off score. As shown in Fig. 1I,J, both TGFBR2 and hTERT had predictive values in cervical cancer, with the maximum area under the curve (AUC) reaching 0.630 and 0.652 for TGFBR2 and hTERT abundance, respectively (Fig. 1I,J, red arrows). High level of protein expression was the point in the curve corresponding to the maximum specificity and sensitivity. For TGFBR2 expression, survival had the maximum AUC and a cut-off score of 1.125; for hTERT expression, International Federation of Gynecology and Obstetrics (FIGO) had the maximum AUC, and a cut-off score of 7.29 (see Supplementary Table S2). Therefore, for tumor samples, scores below and above the cut-off score were designated as low expression and high expression, respectively.

### Major clinicopathological features, and the association of TGFBR2 and hTERT expression patterns with clinicopathologic features

The median age of the cancer patients was 45 years old, ranging from 23 to 78. The mean follow-up time (from beginning of treatment to death or the last follow-up) was 46 months, ranging from 5 to 123. Other clinical data were presented in Table 3.

TGFBR2 and hTERT expression levels were detected in 164 cervical cancer tissues and their clinicopathologic features were summarized in Table 3. Interestingly, the results showed that low TGFBR2 expression was significantly negatively correlated with FIGO stage, differentiation grade, pelvic lymph node metastasis, recurrence, and vital status (P < 0.05), but no significant association was observed between TGFBR2 expression and other clinicopathologic features, such as age, abortion, menopausal status, tumor size, histological type, vaginal invasion, parametrial infiltration, and adjuvant radiotherapy (P > 0.05). In contrast, high expression of hTERT was positively correlated with FIGO stage, differentiation grade, and recurrence (P < 0.05), but not with other clinicopathologic features (P > 0.05).

Patterns of dual TGFBR2/hTERT protein expression in the 164 cases of cervical cancer included: TGFBR2^high^/hTERT^high^ in 17 (10.4%) cases, TGFBR2^high^/hTERT^low^ in 68 (41.5%) cases, TGFBR2^low^/hTERT^high^ in 24 (14.6%) cases, and TGFBR2^low^/hTERT^low^ in 55 (33.5%) cases. It is interesting that TGFBR2^high^/hTERT^high^ and TGFBR2^low^/hTERT^low^ did not show significant association with FIGO stage, pelvic lymph node metastasis, recurrence, or vital status. However, TGFBR2^high^/hTERT^low^ was significantly correlated with FIGO stage and vital status (P < 0.05, Table 4), but not with the differentiation grade, pelvic lymph node metastasis, and recurrence (P > 0.05, Table 4). Compared with others, TGFBR2^low^/hTERT^high^ was significantly correlated with FIGO stage, differentiation grade, pelvic lymph node metastasis, recurrence, and vital status (P < 0.05, Table 4).

### Association between clinicopathologic features, TGFBR2, hTERT, and survival: univariate and multivariate survival analyses

In the 164 cervical cancer cases, the 1-, 3- and 5-year survival rates were 95.7%, 80.4%, and 69.5%, respectively. Univariate analysis revealed that a number of traditional factors, such as FIGO stage, differentiation grade and lymph node metastasis, can predict cervical cancer prognosis, and TGFBR2 expression and different dual TGFBR2/hTERT expression (P < 0.05; see Supplementary Table S3) were also shown as a prediction of cervical cancer prognosis. The survival curves of single TGFBR2 or hTERT, and dual TGFBR2/hTERT were shown in Fig. 1K. These factors were further integrated into a multivariate analysis using Cox proportional hazards analysis method. It was identified that pelvic lymph node metastasis and TGFBR2 expression were independent prognostic factors in cervical cancer (Table 5).

Multivariate analysis were used to find the most risky factor among single expression of TGFBR2, hTERT, and different patterns of dual TGFBR2/hTERT expression. Table 5 showed that the patients with TGFBR2^low^ tumors had significantly higher cumulative incidence of cancer-related death than patients with TGFBR2^high^ tumors (HR = 1.704, P = 0.021). The incidence of death was not different between patients with hTERT^high^ or hTERT^low^ (HR = 1.390, P = 0.181). However, the combination of low TGFRB2 expression and high hTERT expression resulted in a statistically significant increase in the incidence of death than other patterns of dual TGFBR2/hTERT expression (HR = 1.892, P = 0.020). Moreover, when comparing TGFBR2^low^/hTERT^high^ with TGFBR2^high^/hTERT^low^, the incidence of death was much higher (HR = 2.209, P = 0.011). Furthermore, when comparing TGFBR2^high^/hTERT^high^, TGFBR2^high^/hTERT^low^ and TGFBR2^low^/hTERT^low^ with others, no significant difference was found in the incidence of death (HR = 0.723, P = 0.447; HR = 0.636, P = 0.061 and HR = 0.832, P = 0.442, respectively). These results indicate that except the TGFBR2^low^/hTERT^high^ phenotype, other patterns of dual TGFBR2/hTERT expression are protection factors for cervical cancer.

### Correlation between TGFBR2 and hTERT expression levels

The Spearman correlation coefficient (r) for TGFBR2 and hTERT expression in normal, CIN-I, CIN-II, CIN-III and cancer samples was −0.734 (P = 0.01), −0.232 (P = 0.10), 0.391 (P = 0.10), 0.512 (P = 0.01), −0.120 (P = 0.13), respectively, indicating that the correlation between TGFBR2 and hTERT is distinct at different periods of progression of cervical cancer (Tables 6 and 7).

### Downregulation of TGFBR2 promotes cell growth, G1/S transition, cell invasion but reduces cell apoptosis

**Figure 2:**
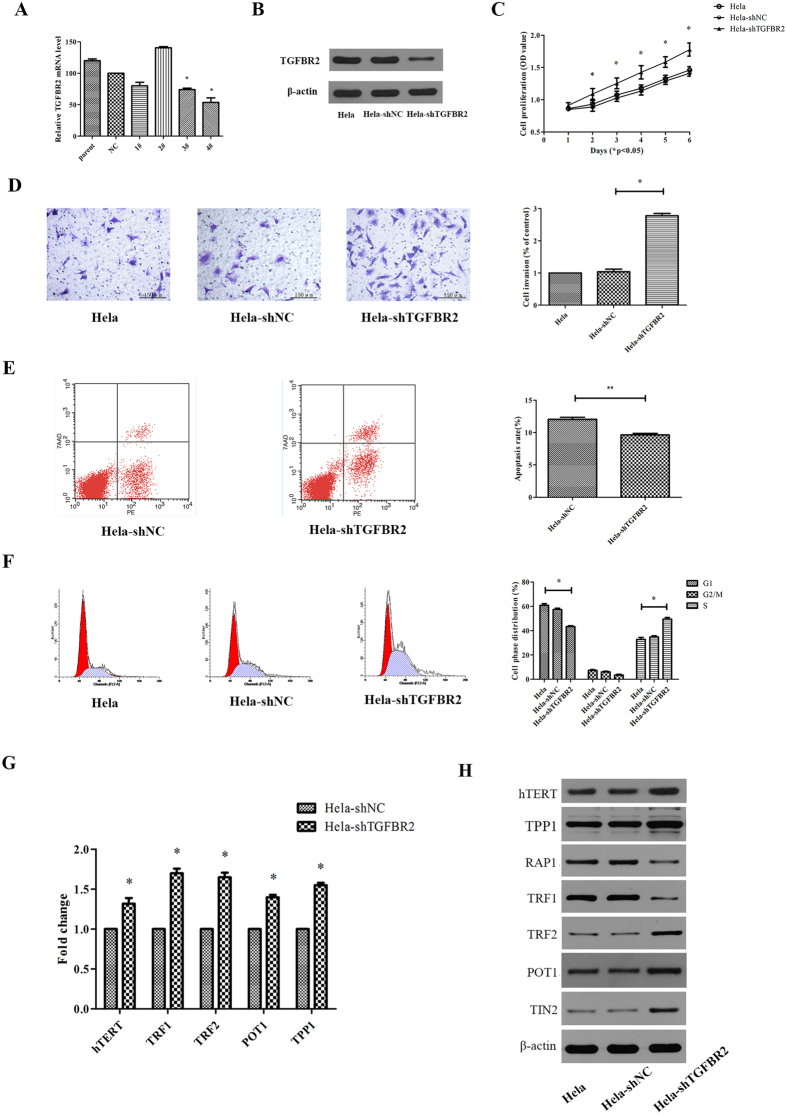
The effectiveness of TGFBR2 knockdown on cell phenotype, shelterins and hTERT. (**A**) Hela cells were transfected with sh*TGFBR2* 1–4#, shNC or no transfection. After 24 h transfection, real-time PCR was used to verify the knockdown effectiveness on Hela cells. (**B**) Hela cells were transfected with *shTGFBR2* 4#, shNC or no transfection. After 72 h transfection, western blot was used to detect the protein level of TGFBR2. (**C**) The cell proliferation after Hela cells transfected with sh*TGFBR2* 4#, shNC or no transfection. (**D**) The cell invasion after Hela cells transfected with sh*TGFBR2* 4#, shNC or no transfection. (**E**) The cell apoptosis after Hela cells transfected with sh*TGFBR2* 4#, shNC. (**F**) The cell cycle after Hela cells transfected with sh*TGFBR2* 4#, shNC or no transfection. (**G**) The *hTERT* and shelterins mRNA level after Hela cells transfected with sh*TGFBR2* 4# or shNC. (**H**) The hTERT and shelterins protein level after Hela cells transfected with sh*TGFBR2* 4#, shNC or no transfection. The uncropped blots details are provided in Supplementary Fig. S4A,B. *P < 0.05, **P < 0.01.

TGFBR2 is closely related to clinicopathologic features and survival. Thus, we verified these results in Hela cells by knockdowning *TGFBR2*. After transfecting plasmids containing the ShRNA duplexes (designed against *TGFBR2*), real-time PCR and western blot were used to analyze TGFBR2 expression. Among Hela, Hela-shNC and Hela-shTGFBR2 cells, the expression of TGFBR2 was reduced in Hela-shTGFBR2 cells both at mRNA (Fig. 2A) and protein levels (Fig. 2B) (P < 0.05). However, Hela-shNC and Hela cells showed subtle change in *TGFBR2* expression. The results indicated that the sequence, especially TGFBR2 knockdown, was successful. Figure 2C shows that TGFBR2 downregulation promoted the proliferation of Hela cells. Transwell results indicated that the invasive ability of Hela-shTGFBR2 cells was significantly higher than that of Hela-shNC cells (Fig. 2D, P < 0.05). The percentage of apoptotic cells for Hela-shNC and Hela-shTGFBR2 was 12.05 ± 0.55 and 9.64 ± 0.41, respectively. It suggested that TGFBR2 knockdown reduced apoptosis in Hela cells (Fig. 2E, P < 0.05). Figure 2F shows that compared to Hela-shNC cells, TGFBR2 depletion had less effect on the proportion of cells in the G2/M phase, but it significantly reduced the number of cells in the G1 phase and increased the proportion of S phase.

### Downregulation of TGFBR2 enhances telomere integrity by increasing hTERT and shelterin proteins expression

Telomere stability correlates with the amount of shelterin and hTERT which consists of telomerase. We determined the effect of knockdown of *TGFBR2* on telomeres and found that knockdown of *TGFBR2* significantly increased the gene expression levels of *hTERT, TRF1, TRF2, POT1*, and *TPP1* (P < 0.05, Fig. 2G). Furthermore, the protein levels of hTERT, TRF2, POT1, TPP1 and TIN2 were much higher expressed but TRF1 and RAP1 were much lower expressed compared to those in Hela-shNC cells, while Hela and Hela-shNC cells (Fig. 2H) exhibited no difference.

### TGFBR2 overexpression together with hTERT inhibition promotes the inhibition of Hela cells growth

**Figure 3:**
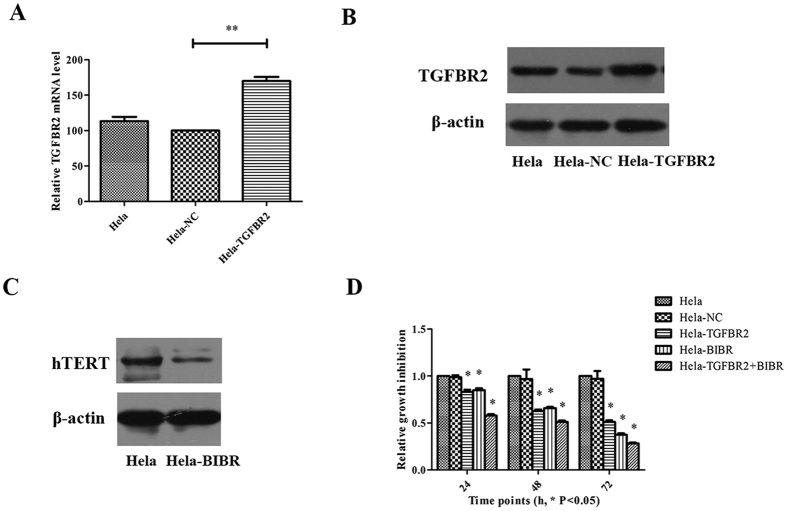
Overexpression of TGFBR2 alone or combined with BIBR1532 on cell proliferation. (**A**) Hela cells were transfected with plasmids containing *TGFBR2* whole coding sequence, negative control (NC) or no transfection. After 24 h transfection, real-time PCR was used to verify the overexpressed effectiveness on Hela cells. (**B**) Hela cells were transfected with plasmids containing *TGFBR2* whole coding sequence, NC or no transfection. After 72 h transfection, western blot was used to detect the protein level of TGFBR2. (**C**) Hela cells were treated with 50 μM BIBR1532. After 48 h incubation, western blot was used to detect the protein level of hTERT. (**D**) TGFBR2 and BIBR 1532 alone or combined in Hela cells, cck-8 were used to analyze the cell proliferation in five groups as follow: Hela, Hela-NC, Hela-TGFBR2, Hela-BIBR1532 and Hela-TGFBR2 + BIBR1532. The uncropped blots details are provided in Supplementary Fig. S4C. *P < 0.05, **P < 0.01.

To further verify the results observed above in this study, after transfection of plasmids containing the whole *TGFBR2* coding sequences, real-time PCR and western blot were performed to analyze TGFBR2 expression. The expression of TGFBR2 was increased in Hela-TGFBR2 cells at both mRNA (Fig. 3A) and protein levels (Fig. 3B) (P < 0.05). However, Hela-NC and Hela cells showed no change in TGFBR2 expression. These confirmed the success of the coding sequence, especially for the *TGFBR2* overexpression. It has been shown that BIBR1532 reduces hTERT expression at the concentration range from 30 to 80 μM^13^,^25^. Therefore, the concentration of 50 μM was used for the follow-up study. It was shown that hTERT knockdown was successful after 48 h of 50 μM BIBR1532 administration (Fig. 3C).

Then, cck-8 was used to detect the cell inhibition in the following five groups, including Hela, Hela-NC, Hela-TGFBR2, Hela-BIBR1532 and Hela-TGFBR2 + BIBR1532. The results (see Fig. 3D) indicate that the overexpression of *TGFBR2* combined with *hTERT* inhibition could effectively inhibit cell growth (Fig. 3D, P < 0.05).

## Discussion

In this study, we demonstrate for the first time that: (1) concomitant underexpression of TGFBR2 and overexpression of hTERT were associated with worse prognosis in cervical cancer; (2) knockdown of TGFBR2 promoted cell growth, cell invasion and G1/S transition; but reduced cell apoptosis; (3) downexpression of TGFBR2 led to higher expression of hTERT, TRF2, POT1, TIN2 and TPP1; (4) overexpression of TGFBR2 combined with BIBR1532 significantly inhibited cell growth.

Abundant studies showed that telomerase expression plays an essential role in cellular immortalization and the malignant transform procession. In cervical cancer transformation, the telomerase activity is negative in normal cervices, while positive in cervical cancers^26^. Some studies on telomerase subunits revealed that *hTR* is not statistically significantly different between normal cervices and cervical cancers^10^. However, other studies showed that *hTERT* mRNA expression is statistically higher in cervical cancers^27^, and seems to be an early malignant event in cervical carcinoma^9^. In line with the results reported by Michael Frost *et al*. in 2000^28^, the present study also showed that hTERT was located at the nucleus in benign tissues, but it gradually transferred into the cytoplasm in the process of malignant transformation. These phenomena suggest that the reduction of hTERT nuclear translocation and the increase of hTERT degradation are associated with malignant transformation in cervical cancer. Moreover, in chronic cervicitis, CIN and cancer tissues, about 81.8%, 94.8% and 95.1% of patients were highly expressed hTERT, respectively. Although these changes have limited use in clinical direction, they might be developed as auxiliary approaches to assist early diagnosis of cervical cancer.

Although hTERT expression has been considered as a diagnostic biomarker of cervical dysplasia and carcinoma^26^, sparse data are available for its correlation with clinicopathologic prognosis and survival in cervical cancer. In the present study, hTERT expression level was correlated with FIGO stage, differentiation grade, and recurrence, but not with pelvic lymph node metastasis or overall survival time. Patients with high hTERT expression seem to have a higher risk of death than those with low hTERT expression (HR, 1.45), but this trend was not statistically significant (P = 0.181). These results are similar to those reported by G. Bea A *et al*. in 2001^15^, which showed that the hTERT is not associated with some factors related to progression-free survival, i.e. tumor volume, vascular invasion, and presence of metastatic lymph nodes. However, in cervical cancer cell lines, some studies showed that knockdown of hTERT could inhibit cell growth^11^ and sensitizes cancer cells to ionizing radiation and chemotherapy^29^; overexpression of hTERT could increase the cell invasion by upregulate MMP proteins^30^. In this study, our results indicate that the hTERT expression alone had limited prognostic significance, although hTERT has been shown a potential therapeutic target for cervical cancer. These may be because the tumorigenesis is a complex process and the tissue samples from different patients are diverse.

Transforming growth factor beta receptor type II (TGFBR2) is the first protein that binds to transforming growth factor-β (TGF-β) to transduce the TGF-β-mediated growth arrest signal into cytoplasm^16^. Abnormal TGFBR2 expression might lead to unsuccessful cell growth arrest regulated by TGF-β, thereby promoting the cell malignant transformation. Previous studies suggested that low expression of TGFBR2 is correlated with an increased risk of nasopharyngeal^31^, breast^32^, and colon^33^ carcinomas. *TGFBR2* is mutated in colorectal carcinoma^34^, ovarian tumors^35^, and breast cancer^36^. The *TGFBR2* gene is a common target for microsatellite-unstable and microsatellite-stable mutations, which can induce cancer progression^37^. Similarly with other tumors, our study revealed that TGFBR2 was underexpressed in cervical cancer tissue, but highly expressed in chronic cervicitis.

When we focused on TGFBR2 expression in the prognosis of cervical cancer, our results showed that TGFBR2 expression was correlated with FIGO stage, differentiation grade, pelvic lymph node metastasis, and recurrence. Compared with high expression of TGFBR2, low expression of TGFBR2 was associated with a higher incidence of cancer-related death (HR = 1.70) and poor outcomes (P = 0.034). After knock down of TGFBR2 in Hela cells, our study found that the cell proliferation and invasion were significantly increased. Moreover, because the inhibition of G1 cyclin-dependent kinases^38^ is one of the main causes of TGF-β-mediated growth arrest, TGFBR2 underexpression could alleviate the inhibition of G1 phase arrest mediated by TGF-β/Smad pathway and accelerate tumor cancer cell cycle from the G1 phase into the S phase. Actually, in this study, knock down of TGFBR2 could accelerate the G1/S transition but reduce the cell apoptosis in Hela cells. These results indicated that TGFBR2 expression is not only a biomarker of cervical cancer, but also an independent prognostic factor in cervical cancer.

Interestingly, our results showed a positive correlation between TGFBR2^low^/hTERT^high^ tumor status, the FIGO stage, differentiation grade, pelvic lymph node metastasis, and recurrence. Moreover, patients in TGFBR2^low^/hTERT^high^ tumor status had a shorter 5-year survival rate when compared to the other groups: TGFBR2^high^/hTERT^high^, TGFBR2^high^/hTERT^low^ and TGFBR2^low^/hTERT^low^ (61.8% vs 81.9%, 70.1%, 72.5%; P = 0.015). Furthermore, patients with TGFBR2^low^/hTERT^high^ had a higher incidence of cancer-related death (HR = 1.892), while the risk of patient death with only high expression of hTERT or low expression of TGFBR2 was lower (HR = 1.390 and 1.704, respectively). Although just 14.6% patients with TGFBR2^low^/hTERT^high^, when comparing this phenotype to TGFBR2^high^/hTERT^low^ phenotype which consists of 41.5% patients and is associated with a favorable prognosis, the incidence rate of death is the highest. These results indicate that concomitant low expression of TGFBR2 and high expression of hTERT might be a more reliable predictor for the prognosis of cervical cancer than low expression of TGFBR2 or high expression of hTERT alone. Predicting the patients with TGFBR2^low^/hTERT^high^ phenotype beforehand and choosing the best target therapeutic strategy might be useful to improve the prognosis and treatment in cervical cancer. Based on the direction identified from these results, more studies may be needed to verify the finding and further explore its values.

Also importantly, we found that TGFBR2 negatively correlated to hTERT in normal samples (r = −0.734, P = 0.01); but gradually shifted to a positive correlation during malignant transformation. In CIN-III samples, TGFBR2 was positively related to hTERT (r = 0.512, P = 0.01) with statistical significance. However, in cancer tissues, TGFBR2 seems negatively related to hTERT, (r = −0.12, P = 0.13). Drabsch Y, *et al*. have reported that TGF-β/SMAD pathway acts as a tumor suppressor at the early stage but functions opposite at late stages^39^. Thus, the role of TGFBR2 may be different at different time points. At the beginning of cervical cells malignant transformation, TGFBR2 is a tumor suppressor and it could repress the hTERT expression, while at the late period of malignant transformation, TGFBR2 could accelerate hTERT expression to complete malignant transformation. When the cervical cells had successful malignant transformation, the TGFBR2 expression showed negatively related to hTERTexpression, despite not being obvious.

In view of the process of tumorigenesis is complex and the patients’ samples shows little uniformity, our further study aimed to verify the correlation of TGFBR2 and hTERT in cervical cancer cells. The results showed that knock down of TGFBR2 resulted in high expression of hTERT. As telomerase and shelterin proteins (TRF1, TRF2, POT1, TPP1, RAP1, and TIN2) can protect telomere (the end of linear chromosomes) from shortened^3^; after knock down of TGFBR2, the results showed that TGFBR2 was negatively related with shelterin proteins in most part. He Li *et al*.^36^ suggested that TGF-β can repress *hTERT* gene expression in human breast cancer cells by downstream Smad3 binding to the *TERT* promoter and repressing *TERT* gene transcription^40^. Another study revealed that this process is related to Smad3 binding to the promoter of c-myc^41^, in which the promoter binds to the *hTERT* promoter to regulate *hTERT* gene expression and repress c-myc gene expression^42^. Moreover, downregulation of telomerase maintains telomeres which is a key factor to induce cell differentiation mediated by TGF-β^22^. Furthermore, TGF-β induced pancreatic tumor cell cycle arrest and cell apoptosis depend on hTERT^22^. Thus, TGFBR2 could negatively regulate hTERT expression indirectly via downstreaming molecular Smad3 transduction of the signal into the nucleus and binding c-myc and hTERT promoter to repress their expression. However, other direct or indirect ways may also exist to modulate this procession, requiring further study in the future.

Most notably, our study revealed that combined TGFBR2 overexpression with hTERT inhibitor BIBR1532, the cell growth is suppressed more efficiently than any single treatment. BIBR1532 (BIBR), a small molecular inhibitor of hTERT, holds a lagging effect on the inhibition of telomerase activity and the decrease of cell proliferation in a variety of tumor cell lines^43^, e.g. chondrosacoma^44^, lung cancer, breast cancer, prostate cancer^43^ and endometrial cancer cell lines^13^. In addition, the survival curve showed that the survival of TGFBR2 high or low expression is different almost from 50 months, but for that of hTERT is almost after 60 months. Thus, this result indicates that the combination of TGFBR2 overexpression and BIBR1532 might enhance the cell inhibition and alleviate the lagging effect which is induced by single BIBR1532. However, further studies should be needed to verify this novel treatment and evaluate its safety via finding a suitable model organism and comprehensive testing.

In summary, our study verified the correlations of TGFBR2 and hTERT *in vitro* and suggests that TGFBR2 and hTERT expression may be used as a diagnostic biomarker for cervical dysplasia and carcinoma. Low expression of TGFBR2 (but not hTERT) seems to be related to poor prognosis for cervical cancer patients, while the concomitant low expression of TGFBR2 and high expression of hTERT, may be considered as a better survival biomarker than low expression of TGFBR2 alone. The combination of TGFBR2 overexpression and hTERT inhibition led to a relatively stronger inhibition of cell growth. Our results suggest a possibility that simultaneous application of TGFBR2 agonist and hTERT inhibitors may be developed as a therapeutic strategy for treating cervical cancer, especially in the patients with TGFBR2^low^/hTERT^high^ expressions.

## Materials and Methods

### Ethics

This study followed the principles of the Helsinki Declaration and was approved by the Ethics Committee of Zhongnan Hospital, Wuhan University, Wuhan, Hubei, China (Permit Number: 2014054). Verbal informed consent was obtained from all patients.

### Patients and tumor samples

We gathered 167 cervical cancer cases at the Zhongnan Hospital of Wuhan University (Wuhan, Hubei, China) from January 2003 to July 2012. All cases were selected based on the following criteria: patients had a pathologically confirmed diagnosis of cervical carcinoma, and primary radical resected specimens were available for tissue microarray (TMA); patients were excluded if they underwent palliative resection, neoadjuvant chemotherapy, or radiotherapy or had a second primary tumor or previous malignant disease. The International Federation of Gynecology and Obstetrics (FIGO) staging system was used to clarify the clinical stage of cervical cancer patients. The characteristics of the patients were obtained from medical records. It should be mentioned that all enrolled patients had FIGO stage I or II disease. According to the National Comprehensive Cancer Network (NCCN) Guidelines, most patients underwent radical hysterectomy with pelvic lymph node dissection. However, those susceptible to an increased risk of recurrent disease were treated with adjuvant radiotherapy or platinum-based concurrent chemoradiation. The patients were followed-up every 1–3 months until data were censored.

### Tissue microarray construction

The TMA was conducted to increase the sample size and to test the validity of IHC results. Through testing IHC results by TMA, the reproducibility of this research has been examined. Two slides of tumor TMAs were constructed from 167 paraffin-embedded tissue specimens. All paraffin blocks were provided by the Department of Pathology, Zhongnan hospital. Briefly, an institutional pathologist defined representative tumor areas by reviewing hematoxylin and eosin-stained full-face sections from all cases. For each specimen, three or four 1.0-mmdiameter sections were circled. Xinchao company, Shanghai, China, helped complete construction of the TMAs. Additionally, 124 tissues from cervical intraepithelial neoplasia (CIN) cases and 14 normal cervical tissues were purchased from Fanpu Biotech, Inc. (Guilin, Guangxi, China).

### Immunohistochemistry (IHC) and evaluation

IHC was performed to examine TGFBR2 and hTERT expression in cervical tissues by using primary antibodies against TGFBR2 (1:100 dilution; ab61213, Abcam, USA) and hTERT (1:25 dilution; ab183105, Abcam, USA), according to the reported procedures^45^. A semi-quantitative estimate was composed based on the staining intensity and the extent of stained cells; the evaluation criteria were as follows: intensity [categorized as 0 (no staining), 1 (weak staining, light yellow), 2 (moderate staining, yellowish brown), or 3 (strong staining, brown)] and the percentage of positively stained cells [scored as 0 (0–5% positive), 1 (6–25%), 2 (26–50%), 3 (51–75%), or 4 (>75%)]. Finally, intensity was multiplied by the percentage of positively stained cells. The results were assessed independently by two investigators who were blinded to the patient’s clinicopathological outcomes. When the investigators originally proposed different results, they reached a consensus. The average result of the two independent investigators was the final reported staining score. Both scores of all the samples were clarified into four categories: 0–1 represented negative expression (−), 2–4 weak expression (+), 5–8 moderate expression (++), and ≥9 high expression (+++).

### Selection of cut-off score

In order to examine the relationship between the expression of these proteins and the clinicopathological features, the cut-off scores for “high expression” of *TGFBR2* and *hTERT* were defined using a receiver operating characteristic (ROC) curve analysis. First, we dichotomized the clinicopathological characteristics into the following groups: FIGO stage classification (IB versus II), tumor size classification (<4 cm versus ≥4 cm), pelvic lymph node metastasis classification (Yes versus No), vaginal invasion (Yes versus No), parametrial infiltration (Yes versus No), recurrence status (Yes versus No), and survival status (death due to cervical cancer versus censored). Second, the expression scores for TGFBR2 and hTERT were used in the ROC analysis. The cut-off score was the point on the curve at which sensitivity and specificity were maximal.

### Cell lines, plasmids, and transfection

The human cervical cancer cells, Hela, were acquired from the Shanghai Institute of Health Sciences (Shanghai, China). The cells were cultured in minimum essential medium (MEM; Thermo fisher, USA), supplemented with 10% fetal bovine serum, and incubated under 5% CO_2_ at 37 °C.

The plasmids contained the ShRNA duplexes were designed against *TGFBR2* (NM_003242) with the following sequences: 5′-AGAACACTTCAGAGCAGTT-3′, and the negative control sequences: 5′-TTCTCCGAACGTGTCACGT-3′ were bought from the interfere with the plasmid library of Genechem (Shanghai, China).The plasmids contained the whole TGFBR2 coding region were bought from the plasmid library of Vigene (Shangdong, China).

Cells were plated in two 6-well plate culture flasks with 3 × 10^5^ per well. After 24 h, we used Turbfect (Thermo fisher, USA) as a transfection reagent according to the manufacturer’s instructions. Finally, after 36 h from the transfection, the cells from 1–4# *TGFBR2* knock down groups, TGFBR2 overexpressed groups, one negative group, and their parental groups, were collected to perform RNA extraction. After 72 h from the transfection, each group of cells were collected to perform protein extraction.

### RNA extraction and quantitative real-time PCR

Total RNA was isolated from cell lines using the TRIzol reagent (Biosharp, China) according to the manufacturer’s protocol. cDNA was synthesized from no more than 5 μg of total RNA by the RecertAidTM First-Strand cDNA Synthesis Kit (Fermentas, Canada) at 42 °C for 10 min, followed by 75 °C for 2 min. Real-time PCR was performed with SYBR Premix Ex TaqTM (Takara, Japan) in a 25 μL reaction volume (12.5 μL SYBR Premix Ex Taq, 200 mM forward and reverse primers, 10 μl H_2_O, and 1.5 μl cDNA template) on an MJ Opticon Monitor Chromo4TM instrument (Bio-Rad, CA). The following protocol was used for real-time PCR-amplification: preincubated at 94 °C for 2 min followed by 40 cycles of 94 °C for 30 sec, 60 °C for 30 sec and 72 °C for 45 sec, and then 72 °C for 10 min. Sangon Biotech (Shanghai, China) helped with designing and synthesizing all the primers as follows: *GAPDH* (forward primer 5′-TGGAAGGACTCATGACCACA-3′, reverse primer 5′-TTCAGCTCAGGGATGACCTT-3′); *TGFBR2* (forward primer 5′-CAACATCAACCACAACACAGAG-3′, reverse primer 5′-CCGTCTTCCGCTCCTCAG-3′); *hTERT* (forward primer 5′-GCGTTTGGTGGATGATTTCT-3′, reverse primer 5′-GCGGTTGAAGGTGAGACTG-3′); *TPP1* (forward primer 5′-ATCTCGAAGTATGCCTGGCCGCTGTCAGAGTG-3′, reverse primer 5′-AGCGGCCGCTATCACATCGGAGTTGGCTCAGAC-3′); *POT1* (forward primer 5′-TCAGATGTTATCTGTCAATCAGAACCT-3′, reverse primer 5′-GTTGACATCTTTCTACCTCGTATAATGA-3′); *TRF1* (forward primer 5′-GCTTGCCAGTTGAGAACGATA-3′, reverse primer 5′-AGGGCTGATTCCAAGGGTG-3′); *TRF2* (forward primer 5′-TCCCAAAGTACCCAAAGGC-3′, reverse primer 5′-ACTCCAGCCTTGACCCACTC-3′). Data were analyzed by the 2-DDCt method.

### Western Blot Analysis

Cultured cells were rinsed twice with pre-cold phosphate-buffered saline (PBS) and mixed with 250 μL of lysis buffer (Beyotime biotechnology, China) and 1 mM PMSF (Biosharp, China). After 10 min on the ice, a scraper was used to transfer the cell lysate into an Eppendorf tube. The cells were ultrasonicated 3 times for 30 secon the ice and then centrifuged at 12,000 rpm for 10 min. Subsequently, the supernatant was collected into another Eppendorf tube and a BCA protein assay kit (Beyotime biotechnology, China) was performed to calculate the protein concertration, followed by the incubation of the samples in boiled water for more than 5 min. These samples were separated by 10% SDS-PAGE and transferred to PVDF membranes. After blocking with 5% skim milk in TBST, the membranes were incubated with primary antibodies, against human TGFBR2 (1:1000 dilution; ab61213, Abcam, USA), β-actin (1:10000 dilution; TDY051, TDY, China), hTERT (1:1000 dilution; ab183105, Abcam, USA), PTOP (1:1000 dilution; ab57595, Abcam, USA); TRF1 (1:1000 dilution; ab10579, Abcam, USA); TRF2 (1:1000 dilution; ab13579, Abcam, USA); POT1 (1:1000 dilution; proteintech 10581-1-AP); TIN2 (1:1000 dilution; ab197894, Abcam, USA); RAP1 (1:1000 dilution; ab47234, Abcam, USA) at 4 °C overnight, and then incubated with Horseradish peroxidase-conjugated secondary antibody diluted at 1:20,000, after which the specific bands were visualized by ECL (Advansta, USA). Autoradiographs were recorded onto X-ray films (Eastman kodak Co, USA). Finally, the ImageJ code was used to analyse the density of bands in the resulting films.

### Cell proliferation assay

Cells were plated in 6-well plate culture flasks. After 24 h from the transfection, the cells were diluted with minimum essential medium containing 10% fetal bovine serum and then were seeded at 2*10^3^ cells/well in 96-well plates and cultured in 100 μL culture medium. It should be mentioned that six identical wells were used for each sample. After 24 h, BIBR 1532 was added into the Hela-BIBR1532 and Hela-TGFBR2 + BIBR1532 group, respectively. At different time points, 10 μL of CCK-8 was added to each well, and the plates were incubated at 37 °C for 2 h. The absorbance of each well was then read at 450 nm using a 96-well plate reader. Each experiment was performed at least three times in triplicate wells.

### Cell invasion assay

Transwell invasion assay was used to analyze the ability of Hela cells passing through matrigel-coated filters before or after TGFBR2 knockdown. The assay was measured in a double chamber (Corning Costar, MA) with 8 μm pore size polycarbonate filter. First, the top side of the polycarbonate filter was coated with diluted Matrigel (BD Biosciences, Bedford, MA). Second, Hela-NC and Hela-sh groups (2 × 10^4^ cells/well) were seeded on the upper portion chamber with serum-free media and incubated for 24 h at 37 °C, respectively; medium with 10% FBS was introduced to the lower chamber as chemo attractant. Third, the cells from the upper surface of the membrane were removed by a cotton swab and the cells from the lower surface of the membrane were stained with 0.1% Crystal violet and fixed with methanol. Each experiment was carried out in triplicate.

### Analysis of cell cycle and apoptosis by flow cytometry

Cells were filed in 70% ethanol overnight, and then treated with RNase for 20 min before addition of 5 mg/ml propidium iodide. The cell cycle was analyzed by flow cytometry (Beckman Coulter, USA). Apoptosis was performed using an Annexin V-PE Apoptosis Analysis kit (Sungene Bio, China) according to the manufacturer’s instructions. Fluorescence was measured using a flow cytometer and the data were analyzed using Cell Quest software. All samples were assayed in triplicate.

### Statistical analyses

Statistical analysis was carried out by means of SPSS (version 18; SPSS Inc., Cary, NC, USA). The Kruskal-Wallis H test was used to analyze *TGFBR2* and *hTERT* expression with cancer progression. Significant clinicopathological differences between patients having high and low TGFBR2 and hTERT expression were analyzed by the Chi-squared testing. A survival analysis was performed by the Kaplan-Meier method and the log-rank testing. The Cox proportional hazards model was used to conduct a multivariate analysis and to identify independent prognostic factors. The correlation between the two proteins was analyzed by Spearman coefficients. For the cell-line experiment, the data obtained from Mann-Whitney U test were analyzed using GraphPad Prism 5.0 software. Difference was regarded as statistically significant when P < 0.05 (two-tails).

## Additional Information

**How to cite this article:** Yang, H. *et al*. Concomitant underexpression of TGFBR2 and overexpression of hTERT are associated with poor prognosis in cervical cancer. *Sci. Rep.* **7**, 41670; doi: 10.1038/srep41670 (2017).

**Publisher's note:** Springer Nature remains neutral with regard to jurisdictional claims in published maps and institutional affiliations.

## Supplementary Material

Supplementary Information

## Figures and Tables

**Table 1:** The expression of TGFBR2 in different cervical lesions.

Cervical specimen	Case	The degree of immunoreactivity (%)				H	P-value
		(−)	(+)	(++)	(+++)		
chronic cervicitis^a^	11	3 (27.3)	3 (27.3)	4 (36.4)	1 (9.1)		
CINI^b^	51	10 (19.6)	27 (52.9)	12 (23.5)	2 (3.9)		
CINII^c^	19	5 (26.3)	10 (52.6)	4 (21.1)	0 (0.0)		
CINIII^d^	27	18 (66.7)	7 (25.9)	2 (7.4)	0 (0.0)		
cervical cancer^e^	164	93 (56.7)	66 (40.2)	4 (2.4)	1 (0.6)	55.608	**0.000**

*^,a,d^p = **0.034**; ^a,e^p = **0.001**; ^b,e^p = **0.000**.

(−), IHC score of 0–1; (+), IHC score of 2–4; (++), IHC score of 5–8; (+++) score of ≥9.

**Table 2:** The expression of hTERT in different cervical lesions.

Cervical specimen	Case	The degree of immunoreactivity (%)				H	P-value
		(−)	(+)	(++)	(+++)		
chronic cervictitis^a^	11	2 (18.2)	8 (72.7)	1 (9.1)	0 (0.0)		
CINI^b^	51	0 (0.0)	27 (52.9)	13 (25.5)	11 (21.6)		
CINII^c^	19	1 (5.3)	4 (21.1)	6 (31.6)	8 (42.1)		
CINIII^d^	27	4 (14.8)	4 (14.8)	8 (29.6)	11 (40.7)		
cervical cancer^e^	164	8 (4.9)	67 (40.9)	73 (44.5)	16 (9.8)	52.659	**0.000**

*^,a,d^p = **0.001**; ^a,e^p = **0.014**; ^b,e^p = **0.004**.

(−), IHIHC score of 0–1; (+), IHC score of 2–4; (++), IHC score of 5–8; (+++) score of ≥9.

**Table 3:** Association of different TGFBR2, hTERT expressions and clinicopathological characteristics in cervical cancer patients.

Characteristic	n(%)164	TGFBR2 expression		P-value	hTERT expression		P-value
		Low, n(%)(n = 79)	High, n (%)(n = 85)		Low, n(%)(n = 123)	High, n (%)(n = 41)	
**Age, years**				0.919			0.183
<50	121 (73.8)	58 (47.9)	63 (52.1)		94 (77.7)	27 (22.3)	
≥50	43 (26.2)	21 (48.8)	22 (51.2)		29 (67.4)	14 (32.6)	
**Abortion**				0.901			0.765
Yes	117(71.3)	56 (47.9)	61 (52.1)		87 (74.4)	30 (25.6)	
No	47(28.7)	23 (48.9)	24(51.1)		36 (76.6)	11 (23.4)	
**Menopausal**				0.243			0.608
Yes	43 (26.2)	24 (55.8)	19 (44.2)		31 (72.1)	12(27.9)	
No	121 (73.8)	55 (45.5)	66(54.5)		92(76.0)	29(24.0)	
**FIGO stage**				**0.019**			**0.021**
IB	65 (39.6)	24 (36.9)	41 (63.1)		55 (84.6)	10 (15.4)	
IIA and IIB	99(60.4)	55(69.6)	44(51.8))		68(55.3)	31(75.6)	
**Tumor size, cm**				0.714			0.650
<4	73 (44.5)	34 (46.6)	39 (53.4)		56(76.7)	17(23.3)	
≥4	91 (55.5)	45 (49.5)	46 (50.5)		67(73.6)	24(26.4)	
**Histological type**				0.053			0.888
Squamous carcinoma	145 (88.4)	65 (44.8)	80 (55.2)		108(74.5)	37(25.5)	
others	19 (11.6)	13 (68.4)	6 (31.6)		15(78.9)	4 (21.1)	
**Differentiation grade**				**0.036**			**0.000**
G1 (High)	18(11.0)	5(27.8)	13(72.2)		15 (83.3)	3 (16.7)	
G2 (Medium)	68(41.5)	29 (42.6)	39 (57.4)		63 (92.6)	5 (7.4)	
G3 (Low)	78(47.6)	45 (57.7)	33 (42.3)		45 (57.7)	33 (42.3)	
**Pelvic lymph node metastasis**				**0.009**			0.094
Yes	62 (37.8)	38 (61.3)	24 (38.7)		42(67.7)	20(32.3)	
No	102 (62.2)	41 (40.2)	61 (59.8)		81(79.4)	21(20.6)	
**Vaginal invaded**				0.949			0.928
Yes	71 (43.3)	34 (47.9)	37 (52.1)		53(74.6)	18(25.4)	
No	93 (56.7)	45 (48.4)	48 (51.6)		70(75.3)	23(24.7)	
**Parametrial infiltration**				0.700			0.234
Yes	51(31.1)	22 (45.8)	29 (54.2)		36(68.8)	15(31.2)	
No	113 (68.9)	57 (49.1)	56 (50.9)		87(77.6)	26(22.4)	
**Ajuvant radiotherapy**				0.098			0.454
Yes	104 (63.4)	45 (43.3)	59 (56.7)		80(76.9)	24(23.1)	
No	60 (36.6)	34 (56.7)	26 (43.3)		43(71.7)	17(28.3)	
**Recurrence**				**0.030**			**0.021**
Yes	18 (11.0)	13 (72.2)	5 (27.8)		9(50.0)	9(50.0)	
No	146 (89.0)	66 (45.2)	80 (54.8)		114(78.1)	32(21.9)	
**Vital status(at last follow-up)**				**0.008**			0.104
Alive	86 (52.4)	33 (38.4)	53 (61.6)		69(80.2)	17(19.8)	
Dead	78 (47.6)	46 (59.0)	32 (41.0)		54(69.2)	24(30.8)	

**Table 4:** Association of different dual TGFBR2/hTERT protein expressions and clinicopathological characteristics in cervical cancer patients.

Characteristic	n(%)164	TGFBR2^high^/hTERT^high^(n = 17)	others	P-value	TGFBR2^high^/hTERT^low^ (n = 68)	other	P-value	TGFBR2^low^/hTERT^high^ (n = 24)	others	P-value	TGFBR2^low^/hTERT^low^(n = 55)	others	P-value
**FIGO stage**				0.927			**0.024**			**0.039**			0.745
IB	65(39.6)	6 (9.2)	59(90.8)		35(53.8)	30 (46.2)		4 (6.2)	61 (93.8)		20 (30.8)	45 (69.2)	
IIA	54(32.9)	6 (11.1)	48 (88.9)		20(37.0)	34(63.0)		10 (18.5)	44 (81.5)		18 (33.3)	36 (66.7)	
IIIB	45(27.4)	5 (11.1)	40(88.9)		13(28.9)	32(71.1)		10 (22.2)	35 (77.8)		17 (37.8)	28 (62.2)	
**Differentiation grade**				**0.020**			0.198			**0.003**			0.508
G1 (High)	18(11.0)	2 (11.1)	16 (88.9)		11 (61.1)	7 (38.9)		1 (5.6)	17 (94.4)		4 (22.2)	14 (77.8)	
G2 (Medium)	68(41.5)	12 (17.6)	56 (82.4)		27 (39.7)	41 (60.3)		4 (5.9)	64 (94.1)		25 (36.8)	43 (63.2)	
G3 (Low)	78(47.6)	3 (3.8)	75 (96.2)		30 (38.5)	48 (61.5)		19 (24.4)	59 (75.6)		26 (33.3)	52 (66.5	
**Pelvic lymph node metastasis**				0.200			0.124			**0.007**			0.680
Yes	62 (37.8)	4(6.5)	58(93.5)		21(33.9)	41(66.1)		15(24.2)	47 (75.8)		22 (35.5)	40 (64.5)	
No	102 (62.2)	13(12.7)	89(87.3)		47(46.1)	55(53.9)		9 (8.8)	93(91.2)		33 (32.4)	69 (67.6)	
**Recurrence**				0.764			0.212			**0.001**			0.281
Yes	18 (11.0)	1 (5.6)	17 (94.4)		5 (27.8)	13 (72.2)		8 (44.4)	10 (55.6)		4 (22.2)	14 (77.8)	
No	146 (89.0)	16 (11.0)	130 (89.0)		63 (43.2)	83 (56.8)		16 (11.0)	130 (89.0)		51 (34.9)	95 (65.1)	
**Vital status**				0.285			**0.044**			**0.004**			0.542
Alive	86 (52.4)	11 (12.8)	75 (87.2)		42 (48.8)	44 (51.2)		6 (7.0)	80 (93.0)		27 (31.4)	59 (68.6)	
Dead	78 (47.6)	6 (7.7)	72 (92.3)		26 (33.3)	52 (66.7)		18 (23.1)	60 (76.9)		28 (35.9)	50 (64.1)	

**Table 5:** Estimated risk of death associated with TGFBR2 and hTERT positivity in cervical cancer.

Strata	Hazard ratio	Confidence interval (95%)	P-value
TGFBR2 (low vs high)	1.704	1.08–2.68	**0.021**
hTERT (high vs low)	1.390	0.86–2.25	0.181
TGFBR2^high^/hTERT^high^ vs. Others	0.723	0.31–1.67	0.447
TGFBR2^high^/hTERT^low^ vs. Others	0.636	0.40–1.02	0.061
TGFBR2^low^/hTERT^high^ vs. Others	1.892	1.10-3.24	**0.020**
TGFBR2^low^/hTERT^low^ vs. Others	0.832	0.52-1.33	0.442
TGFBR2^low^/hTERT^high^ vs TGFBR2^high^/hTERT^low^	2.209	1.20–4.08	**0.011**

Adjusted for patients at FIGO stage, differentiation, pelvic lymph node metastasis and recurrence.

**Table 6:** Correlation between TGFBR2 and hTERT expressions in chronic cervicitis and CIN tissues.

	Case	hTERT expression n(%)				P-value
		hTERT (−)	hTERT (+)	hTERT (++)	hTERT (+++)	
**Chronic cervicitis**	11	2 (18.2)	8 (72.7)	1 (9.1)	0 (0.0)	**P** = **0.01**
TGFBR2 (−)	3	0 (0.0)	2 (66.7)	1 (33.3)	0 (0.0)	
TGFBR2 (+)	3	0 (0.0)	3 (1.0)	0 (0.0)	0 (0.0)	
TGFBR2 (++)	4	2 (50.0)	2 (50.0)	0 (0.0)	0 (0.0)	
TGFBR2 (+++)	1	1 (1.0)	0 (0.0)	0 (0.0)	0 (0.0)	
**CIN I**	51	0 (0.0)	27 (53.0)	13 (25.5)	11 (21.5)	P = 0.10
TGFBR2 (−)	10	0 (0.0)	5 (50.0)	4 (40.0)	1 (10.0)	
TGFBR2 (+)	27	0 (0.0)	11 (40.7)	6 (22.2)	10 (37.0)	
TGFBR2 (++)	12	0 (0.0)	9 (75.0)	3 (25.0)	0 (0.0)	
TGFBR2 (+++)	2	0 (0.0)	2 (1.0)	0 (0.0)	0 (0.0)	
**CIN II**	19	1 (5.3)	4 (21.1)	6 (31.6)	8 (42.1)	P = 0.10
TGFBR2 (−)	5	1 (20.0)	2 (40.0)	1 (20.0)	1 (20.0)	
TGFBR2 (+)	10	0 (0.0)	2 (20.0)	3 (30.0)	5 (50.0)	
TGFBR2 (++)	4	0 (0.0)	0 (0.0)	2 (50.0)	2 (50.0)	
TGFBR2 (+++)	0	0 (0.0)	0 (0.0)	0 (0.0)	0 (0.0)	
**CIN III**	27	4 (14.8)	4 (14.8)	8 (29.6)	11 (40.7)	**P** = **0.01**
TGFBR2 (−)	18	4 (22.2)	3 (16.7)	7 (38.9)	4 (22.2)	
TGFBR2 (+)	7	0 (0.0)	1 (14.3)	1 (14.3)	5 (71.4)	
TGFBR2 (++)	2	0 (0.0)	0 (0.0)	0 (0.0)	2 (1.0)	
TGFBR2 (+++)	0	0 (0.0)	0 (0.0)	0 (0.0)	0 (0.0)	

(−), IHC score of 0–1; (+), IHC score of 2–4; (++), IHC score of 5 – 8; (+++) score of ≥9.

**Table 7:** Correlation between TGFBR2 and hTERT expressions in cervical cancer tissues.

	Case	hTERT expression n(%)		P-value
		Low (−)	High (+)	
Cancer	164	123 (75.0)	41 (33.3)	**P = 0.13**
TGFBR2 low(−)	79	55 (69.6)	24 (30.4)	
TGFBR2 high(+)	85	68 (80.0)	17 (20.0)	

TGFBR2 (+), IHC score of ≥1.125; hTERT, IHC score of ≥7.29.
