# Supplementary material for: Concomitant underexpression of TGFBR2 and overexpression of hTERT are associated with poor prognosis in cervical cancer
Source: Sci Rep. 2017 Feb 14;7:41670. doi: 10.1038/srep41670 (PMC5307321; doi:10.1038/srep41670)
Supplement: Supplementary Information [file srep41670-s1.pdf]

Concomitant underexpression of TGFBR2 and overexpression of hTERT are associated with poor prognosis in cervical cancer

Hui Yang[1+], Hongyan Zhang[1,2+], Yahua Zhong[1,2], Qiaoli Wang[1], Lei Yang[1,2], Hong Kang[1], Xiaojia Gao[1], Haijun Yu[1,2], Conghua Xie[1,2], Fuxiang Zhou[1,2*], Yunfeng Zhou[1,2]*

[1]Hubei Key Laboratory of Tumor Biological Behavior, Hubei Cancer Clinical Study Center, Zhongnan Hospital, Wuhan University, Wuhan, China;

[2]Department of Radiation Oncology & Medical Oncology, Zhongnan Hospital, Wuhan University, Wuhan, P.R. China

*Correspondence to: Fuxiang Zhou and Yunfeng Zhou, MD, PhD, Department of Radiation Oncology & Medical Oncology, Zhongnan Hospital, Wuhan University, 169 Donghu Road, Wuhan 430071, China; Fax: +86 27 6781 2889; Email: fxzhou@163.com; yfzhouwhu@163.com

[+]These authors contributed equally to this work

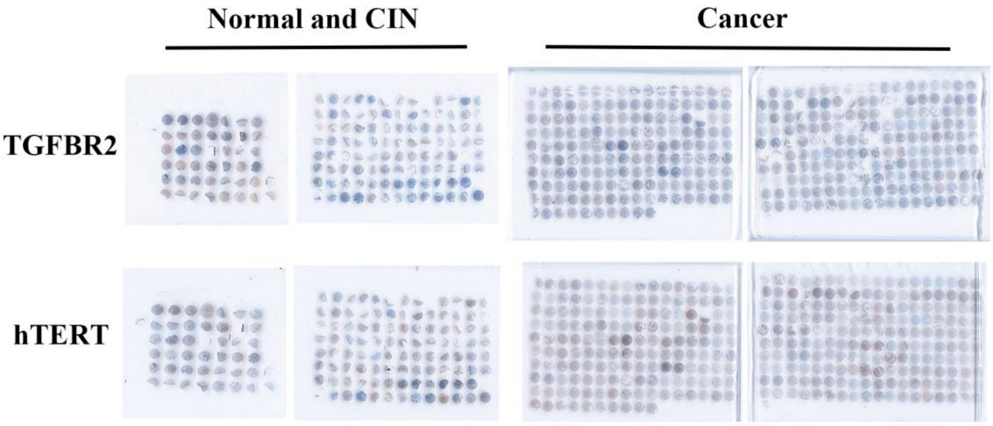

Supplementary Fig. S1. The results of tissue microarray and immunostaining.

Feature	TGFBR2			hTERT		
	**cut off**	**P value**	**AUC**	**cut off**	**P value**	**AUC**
**FIGO stage**	0.875	0.685	0.520	**7.29**	**0.001**	**0.652**
**Tumor size**	1.125	0.899	0.506	3.29	0.285	0.451
**lymph node metastasis**	0.630	0.850	0.509	3.71	0.355	0.542
**Vaginal invaded**	2.125	0.632	0.522	7.13	0.319	0.545
**Parametrial infiltration**	4.250	0.470	0.466	5.13	0.242	0.555
**Recurrence**	3.250	0.069	0.382	3.88	0.057	0.485
**Survival status**	**1.125**	**0.004**	**0.630**	8.75	0.714	0.517

Supplementary Table S2. According to ROC analysis, the cutoff score of TGFBR2 and hTERT expression for each clinicopathological feature were shown below.

Characteristic	n(%)164	Five-year survival rate,%	χ2	P-value
**Age, years**				
<50	121(73.8)	73.0	0.741	0.389
≥50	43(26.2)	64.8		
**Abortion**				
Yes	117(71.3)	72.3	1.198	0.274
No	47(28.7)	67.6		
**Menopausal**				
Yes	43(26.2)	62.8	2.298	0.130
No	121(73.8)	73.8		
**FIGO stage**				
Ⅰ B	65(39.6)	87.7	20.806	**0.000**
Ⅱ A	54(32.9)	58.7		
Ⅱ B	45(27.4)	77.8		
**Tumor size, cm**				
<4	73(44.5)	73.1	0.000	0.997
≥4	91(55.5)	68.9		
**Histological type**			1.535	0.215
Squamous carcinoma	145(88.4)	72.6		
other	19(11.6)	57.0		
**Differentiation grade**				
G1(High)	18(11.0)	75.0	11.507	**0.003**
G2 (Medium）	68(41.5)	81.4		
G3 (Low)	78(47.6)	61.3		
**Pelvic lymph node metastasis**			14.541	**0.000**
Yes	62(37.8)	60.4		
No	102(62.2)	81.2		
**TGFBR2 expression level**			4.470	**0.034**
high	85(51.8)	72.4		
low	79(48.2)	69.2		
**hTERT expression level**			2.319	0.128
high	41(25.0)	70.0		
Low	123(75.0)	63.5		
**Dual TGFBR2/hTERT expression level**			10.450	**0.015**
$TGFBR2^{high}/hTERT^{high}$	17(10.4)	81.9		
$TGFBR2^{high}/hTERT^{low}$	68(41.5)	70.1		
$TGFBR2^{low}/hTERT^{high}$	24(14.6)	61.8		
$TGFBR2^{low}/hTERT^{low}$	55(33.5)	72.5		

Supplementary Table S3. Univariate analysis of factors regarding overall survival.

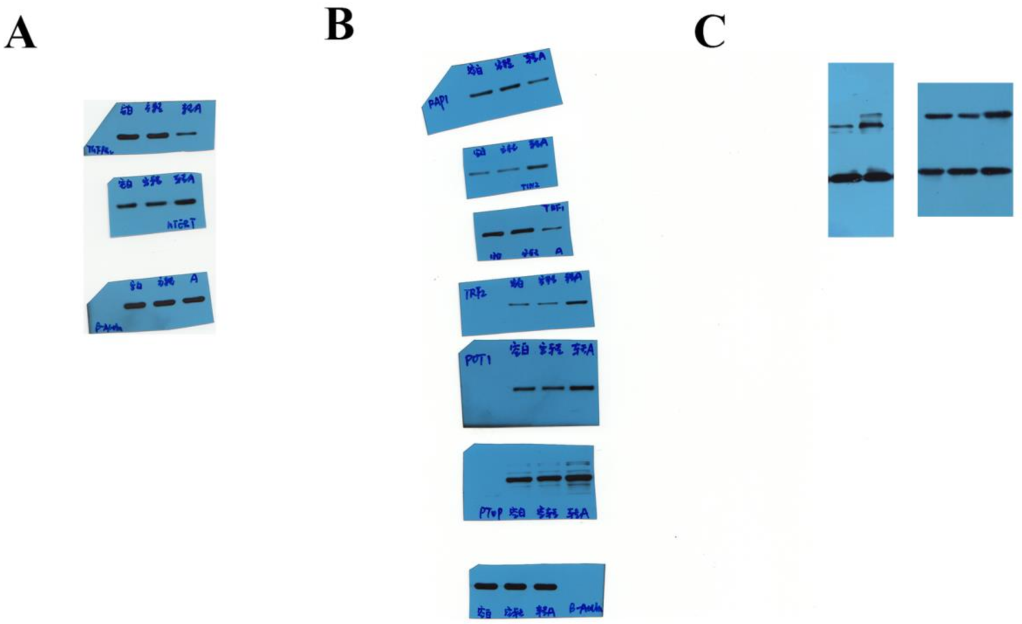

Supplementary Fig. S4. A. The uncropped bolts details in figure 2A. B. The uncropped bolts details in figure 2H. C. The uncropped bolts details in figure 3.
